# Economic Evaluation of Home Chest Radiography for Patients With Limited Mobility in Greece: A Modeled Cost-Minimization Analysis From a Limited Societal Perspective

**DOI:** 10.7759/cureus.115699

**Published:** 2026-09-03

**Authors:** Panagiotis P Bidikoudis, Konstantinos Athanasakis, Christos Michail, Georgios Dounias

**Affiliations:** 1 Department of Radiodiagnostics, University of West Attica, Athens, GRC; 2 Department of Radiodiagnostics, General Hospital of Nikaia-Piraeus, Piraeus, GRC; 3 Department of Public Health Policy, University of West Attica, Athens, GRC; 4 Department of Biomedical Engineering, University of West Attica, Athens, GRC; 5 Department of Occupational and Environmental Health and Industrial Hygiene, University of West Attica, Athens, GRC

**Keywords:** chest x-ray, cost-minimization analysis, greece, health economics, home radiography, limited societal perspective, mobile radiography, patient transport, teleradiology

## Abstract

Introduction

Home radiography is an alternative diagnostic pathway for patients with limited mobility who would otherwise need to be transported to a hospital facility for chest radiography. Transporting these patients entails costs, attendant time, and organizational burden. The purpose of the present study was to compare the cost of two-view home chest radiography (frontal and lateral views) with that of the hospital pathway through a public hospital Emergency Department (ED) in clinically stable patients who do not require urgent medical assessment and who, in the absence of a home service, would be transferred to the hospital solely for the examination. The primary economic outcome was the difference in cost per completed diagnostic episode.

Methods

A modeled cost-minimization analysis (CMA) was conducted from a limited societal perspective that included examination costs, transportation costs, and the opportunity cost of attendant time. Two pathways were compared: home two-view chest radiography and hospital two-view chest radiography after patient transfer via a public hospital ED, modeled narrowly as a transport-and-radiography pathway rather than a full emergency assessment. The analysis assumed a completed diagnostic-quality examination with a radiologist report in the base case. Publicly advertised Greek home-radiography prices were re-evaluated using explicit comparability criteria, Greek ED time data were incorporated in scenario analyses, and a conservative scenario/threshold analysis evaluated technically unsuccessful home examinations followed by the full hospital pathway.

Results

In the base-case scenario from the limited societal perspective, the cost of the home pathway was estimated at €112.66, while the cost of the hospital pathway was estimated at €135.45. Home radiography was €22.79 less expensive per diagnostic episode. The expanded home-radiography price dataset included 11 observations, with a median of €110, an interquartile range of €100-€120, and an observed range of €50-€140. Greek-informed hospital-time scenarios remained in favor of the home pathway, including a conservative two-hour hospital-attendant-time scenario (incremental cost: 6.07). In a stress test in which a technically unsuccessful home examination was followed by the full hospital pathway, the cost-neutral failure/referral probability was approximately 16.8%.

Conclusions

Under the assumptions of the model, home two-view chest radiography may be a cost-saving alternative for appropriately selected, clinically stable patients with limited mobility in Greece when transportation costs and the economic value of attendant time are taken into account. The findings are exploratory and model-dependent; they support further prospective evaluation using Greek patient-level resource-use and pathway data.

## Introduction

The aging population and the increasing prevalence of chronic diseases have increased the number of patients with severe mobility limitations. For these patients, even a simple chest X-ray may require ambulance transport, an attendant, a hospital stay, and return transport to the home or care facility. This pathway entails not only direct medical costs but also broader societal costs, such as transport costs, attendant time, and organizational burden.

Home or mobile radiography offers an alternative approach to delivering the same diagnostic service. Instead of transporting the patient to the radiology department, the radiography service is delivered at the patient's place of residence. A recent scoping review by Costa et al. on home-based diagnostic imaging services showed that such services can improve accessibility, particularly for older adults, people with disabilities, and other vulnerable groups. The same review highlighted logistics, equipment portability, patient experience, and integration of results into the diagnostic pathway as key implementation factors [1].

The clinical utility of home radiography is supported by the international literature. Raiano et al. described the implementation of home chest radiography during the COVID-19 pandemic and reported that the quality of images acquired at home was comparable to that obtained in hospital settings [2]. The RAD-HOME project investigated the provision of radiology services at home for elderly and vulnerable patients for whom transport to the hospital was functionally burdensome [3].

More recently, Eade et al. evaluated the clinical effectiveness and cost-effectiveness of a home-based mobile radiology service in a regional setting in the United Kingdom. The service was implemented as a six-month pilot program, bringing portable radiology equipment to patients' homes or care facilities and integrating the results into existing hospital systems. The study reported reductions in the need for ambulance transport and Emergency Department (ED) use, supporting the potential for mobile radiography to function as a practical outpatient service [4].

Patient acceptance of mobile radiography is also important. Toppenberg et al., in a randomized study comparing mobile radiography with in-hospital radiography, reported higher levels of satisfaction in the mobile radiography group [5]. 

The time required for the hospital pathway is a critical parameter in the economic evaluation. Plant et al. reported a mean length of stay of 5.15 hours among nursing home residents who were transferred to an ED, underwent X-ray only, and returned to the care unit [6]. In another context, García Santos et al. reported a mean waiting time of 5 hours and 48 minutes for patients with suspected respiratory infection in EDs during a COVID-19 epidemic wave [7].

Greek real-world evidence also demonstrates substantial and heterogeneous ED time. Intas et al. reported a mean waiting time of 164.1 minutes and a mean care time of 41.3 minutes in a large Greek public ED, corresponding to approximately 3.42 hours when the two sequential components are combined [8]. Reid et al. subsequently reported average ED length of stay ranging from 4.5 to 6.2 hours across four Greek public EDs [9]. These Greek cohorts are not restricted to clinically stable X-ray-only patients; therefore, they are used as contextual and scenario evidence rather than as direct substitutes for the more pathway-specific 5.15-hour estimate.

International economic data suggest that mobile radiography can reduce overall costs in selected populations. Kjelle et al. performed a cost analysis of mobile radiography services for nursing home residents in Southeast Norway and reported lower costs when a model combining mobile radiography and hospital services was used [10]. Another study by Kjelle et al. showed that the availability of a mobile radiography service can increase the use of diagnostic imaging by nursing home residents, thereby reducing the access gap compared with the general population [11]. Loeb et al., in a randomized study of nursing home residents with pneumonia or lower respiratory tract infection, showed that an on-site clinical pathway reduced hospitalizations and resulted in cost savings [12].

Despite the international evidence, economic evaluations of home radiography in Greece remain limited. The purpose of the present study is to estimate the difference in cost per completed diagnostic episode between home two-view chest radiography and the hospital pathway through a public hospital ED for clinically stable patients with limited mobility who do not require urgent hospital evaluation and would be transported solely for the radiographic examination. The primary economic outcome is the incremental pathway cost (home minus hospital) per completed diagnostic episode.

## Materials and methods

Study design

The present study was designed as a model-based cost-minimization analysis (CMA). CMA was selected because the modeled comparison is conditional on both strategies achieving the same narrowly defined diagnostic endpoint: a diagnostic-quality two-view chest radiograph with a radiologist’s report [13]. This is a modeling assumption rather than empirically demonstrated clinical equivalence for all patients with mobility limitations or for every technical acquisition protocol. It applies only to appropriately selected, clinically stable patients when the home examination is performed with appropriate digital equipment by appropriately trained personnel and within an organized teleradiology consultation process [2-4,14]. The study did not estimate differential health outcomes, downstream treatment effects, or quality-adjusted life-years; therefore, a cost-effectiveness analysis was not undertaken. Future prospective evaluations could use a cost-consequence framework if repeat imaging, patient experience, downstream care, and other relevant consequences are measured alongside costs.

The two pathways compared were home two-view chest radiography and hospital two-view chest radiography requiring patient transport, with transfer through a public hospital ED used as an explicit base-case organizational scenario. This choice should not be interpreted as a claim that all clinically stable patients with limited mobility in Greece are routinely managed through the ED. Rather, it represents the modeled pathway for patients who, in the absence of a home service, would be transported to a public hospital for radiography. In settings where direct access to a hospital radiology department or an outpatient diagnostic center is available, that route would represent a different comparator and may involve shorter waiting and attendant time. The hospital comparator is interpreted narrowly as a transport-and-radiography pathway rather than as a full emergency clinical assessment. Accordingly, no separate cost for ED clinical assessment, laboratory testing, treatment, or other emergency-department resource use was assigned in the model. Patients requiring immediate medical evaluation, laboratory testing, additional imaging, therapeutic intervention, or potential admission are excluded from the model.

For simplicity and transparency, pathway-level costing was applied instead of full micro-costing. Therefore, home X-ray was valued as an overall service price and was not broken down into individual cost components such as equipment, depreciation, personnel, vehicle, fuel, digital infrastructure, and consumables. The economic evaluation was reported in accordance with the CHEERS 2022 statement [15].

Target population

The target population includes adult patients with limited mobility who require a chest X-ray, are clinically stable, and do not require urgent hospital evaluation. Examples include vulnerable older adults, bedridden patients, patients with neurological, musculoskeletal, or stable cardiorespiratory diseases, and patients for whom transport to the hospital is difficult or particularly burdensome.

Perspective and time horizon

The main analysis was carried out from a limited societal perspective, defined for this model as including the cost of the examination, the cost of patient transport, and the opportunity cost of attendant time. Throughout the manuscript, the term 'limited societal perspective' refers specifically to these included cost categories and should not be interpreted as a comprehensive societal evaluation. Patient time, productivity losses, broader informal-care costs, ED resource burden, and intangible costs were not monetized.

In addition, a secondary analysis was performed from the healthcare system/payer perspective, in which only the direct costs that may be borne by the payer of the service were included.

For this model, a 'complete diagnostic episode' was operationally defined as the period from the request for the chest radiograph until completion of the requested two-view examination and availability of the radiologist's report. It does not include subsequent clinical assessment, treatment, hospitalization, additional diagnostic work-up, or long-term follow-up.

Cost year and data sources

All prices were expressed in 2026 euros. Publicly posted market charges for home X-ray and private ambulance transport were recorded from publicly accessible provider websites. The home-radiography market search was updated in August 2026. These prices were treated as publicly advertised charges rather than as full economic resource costs.

For the revised home-radiography price analysis, predefined comparability criteria were applied. A price observation was eligible for the primary dataset when provider information, online booking information, or direct provider confirmation available to the authors established a home-based digital radiography service comprising two projections (F+P/F+Pr or explicitly up to/two acquisitions) and radiological interpretation/reporting. Information on image/result delivery, geographic coverage, and additional travel or out-of-hours charges was recorded where available. Variable prices or services for which the relevant package could not be established were retained in the supplementary evidence table but excluded from the primary median calculation.

Eleven observations met the operational comparability criteria, with prices of €50, €90, €100, €100, €100, €110, €120, €120, €120, €120, and €140 [16-19,20-26]. The median was €110, the interquartile range was €100-€120, and the observed range was €50-€140. The median value of €110 was therefore retained as the base-case home two-view chest-radiography cost. Because the lowest observation was advertised as a starting price, a separate robustness scenario excluded this observation; the resulting median was €115. Full service-component details and inclusion decisions are presented in Appendix 1.

For the hospital pathway, because detailed procedure-level costing data were unavailable for Greek public hospitals, the direct cost of hospital two-view chest radiography was estimated based on the official National Organization for the Provision of Health Services (EOPYY) reimbursement tariff. Chest X-ray F is reimbursed at €4.05, and chest X-ray P is also reimbursed at €4.05. Therefore, a total proxy cost of €8.10 was used [27]. This input is a reimbursement proxy rather than the full resource cost of operating a public-hospital radiology service.

The cost of transporting the patient to and from the hospital was estimated based on published market charges from private ambulance providers in Attica. The recorded prices were €90 and €110 for transport with a pre-arranged return trip. For the base-case scenario, the average value of the available observations was used, i.e., €100. Published values were used as reported, without additional tax adjustment [28,29].

Attendant time was valued using the opportunity cost method, based on the hourly equivalent of the minimum wage in Greece. Based on a minimum monthly salary of €920 and 173.3 working hours per month, the value of attendant time was estimated at €5.31/hour [30]. Unlike the service and transport inputs, this parameter represents an opportunity-cost valuation rather than a market charge or reimbursement tariff. Because the valuation of attendant time can be debated, an additional scenario assigning zero economic value to attendant time was considered.

The base-case hospital attendant time of 5.15 hours was retained from Plant et al. because that study specifically described nursing-home residents transferred to an ED for X-ray only and subsequently returned to the care unit [6]. The cited value represents patient ED/pathway length of stay rather than directly measured caregiver time. In the present model, it is therefore used as a proxy for the duration during which an attendant would accompany the patient through the in-hospital radiography pathway, not as a direct measurement of Greek caregiver time. The monetary cost of ambulance transport is modeled separately; ambulance travel time and healthcare-professional time are not separately monetized. Because the source reports ED length of stay rather than door-to-door attendant time, this proxy may underestimate total accompaniment time. To address external-validity uncertainty, Greek ED evidence was incorporated in scenario analyses: approximately 3.42 hours based on the combined mean waiting and care times reported by Intas et al. [8], and 4.5 and 6.2 hours corresponding to the lower and upper average ED length-of-stay values reported across four Greek public EDs by Reid et al. [9]. A wider two- to eight-hour hospital-attendant-time range was also used as a deterministic stress test.

Decision tree

The clinically stable patient with limited mobility who requires a two-view chest radiograph and does not require further urgent hospital evaluation may follow one of two pathways. The home pathway includes the cost of the home examination and the attendant's time at home. The hospital pathway includes the cost of the hospital examination, the cost of transportation to and from the hospital, and attendant time during the hospital trip. Under the base-case assumptions of the model, the final result of both strategies is a diagnostic-quality chest radiograph with a radiologist’s report. The base-case tree is intentionally deterministic; possible technically unsuccessful home examinations requiring subsequent hospital referral are evaluated separately through scenario and threshold analyses rather than assigned an unsupported base-case probability. The decision tree is shown in Figure 1.

**Figure 1:**
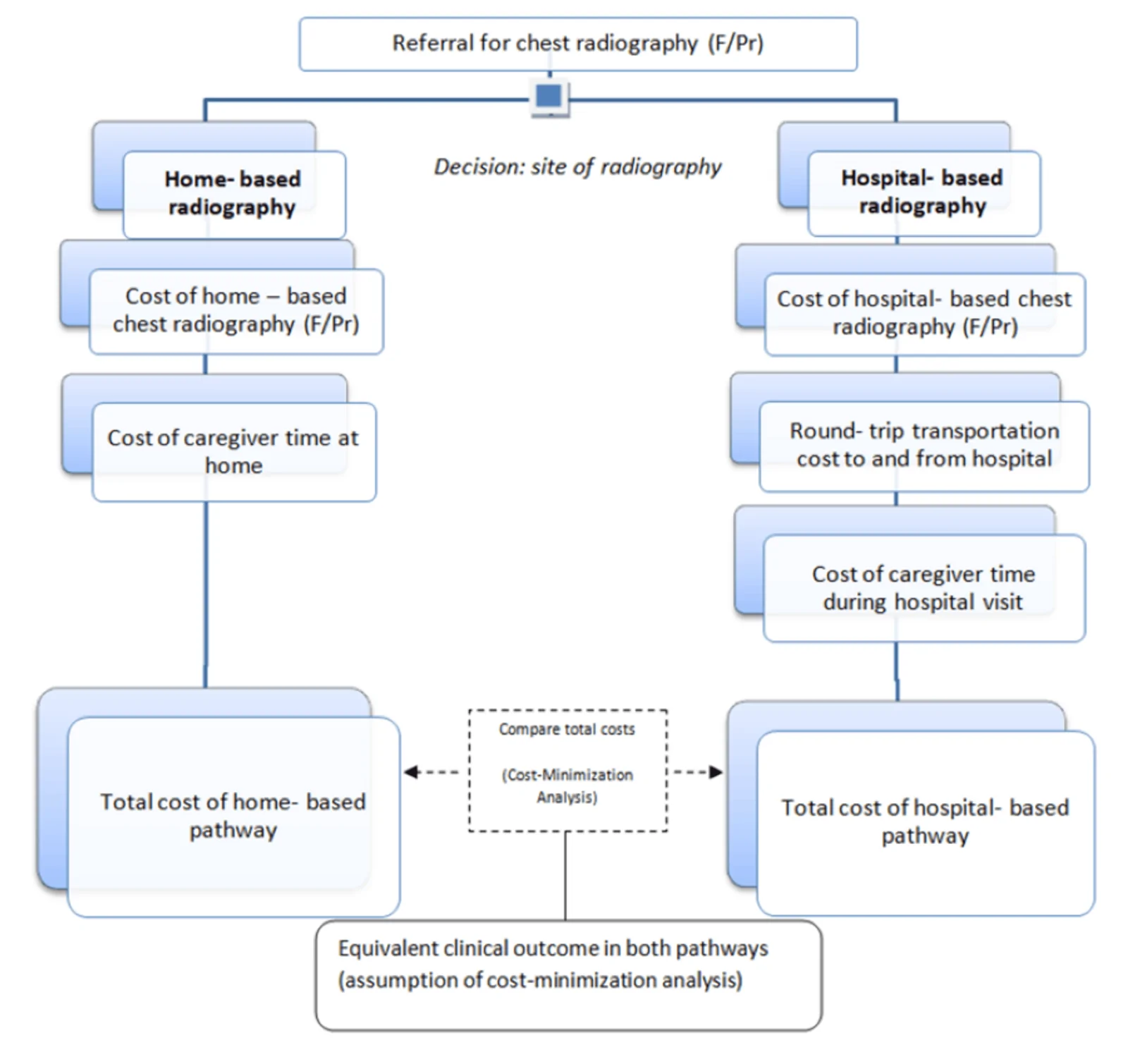
Decision tree of the cost-minimization model The model compares home-based and hospital-based two-view chest radiography pathways for clinically stable patients with limited mobility. Both base-case pathways are assumed to lead to a diagnostic-quality chest radiograph with a radiologist’s report. Technical failure/referral is evaluated separately in scenario analysis.

Cost calculations

The total cost of the home pathway was calculated as: $C_{\mathrm{home}} = C_{\mathrm{xray}} + (T_{\text{escort home}} \times W)$. The total cost of the hospital pathway was calculated as: $C_{\mathrm{hospital}} = C_{\text{hospital xray}} + C_{\mathrm{transport}} + (T_{\text{escort hospital}} \times W)$, where *C *represents cost, *T* represents attendant time, and *W* represents the value of time per hour. The cost difference was calculated as: $\Delta C = C_{\mathrm{home}} - C_{\mathrm{hospital}}$. A negative value of Δ*C* indicates that the home strategy is less costly.

For the technical-failure stress test, the expected cost of initiating the home strategy was calculated as $E(C_{\mathrm{home}}) = C_{\mathrm{home}} + p \times C_{\mathrm{hospital}}$​​​​​​​, where *p* is the hypothetical probability that a technically unsuccessful home examination is followed by the full hospital pathway. This is deliberately conservative because both the initial home-pathway cost and the complete hospital-pathway cost are retained for failed home episodes.

Sensitivity analysis

A deterministic one-way sensitivity analysis was performed by varying one parameter at a time while keeping the others constant. Home X-ray cost was tested across the observed €50-€140 range; transport cost, at €90 and €130; hospital escort time, at two and eight hours; escort hourly value, at €0 and €12/hour; hospital X-ray proxy cost, at €20; and home escort time, at 0.25 and 1.0 hour. Additional scenarios examined the €115 home-price median obtained after excluding the lowest starting-price observation, Greek-informed hospital-time values (3.42, 4.5, and 6.2 hours), and combined easier/difficult hospital pathways. Finally, hypothetical technically unsuccessful home-examination/referral probabilities of 5%, 10%, 15%, and 20% were examined, together with the break-even probability. These probabilities are stress-test values and are not estimates of the true clinical failure rate. The main parameters of the model are presented in Table 1.

**Table 1:** Base-case parameters

Parameter	Base-case value	Rationale
Home-based two-view chest radiography	110 €	Median of 11 operationally comparable observations; IQR €100-€120; observed range €50-€140; Appendix 1
Hospital-based two-view chest radiography	8.10 €	National Organization for the Provision of Health Services (EOPYY) reimbursement proxy; sensitivity value €20
Round-trip patient transport	100 €	Mean of available private ambulance prices; sensitivity values €90 and €130
Escort time, home pathway	0.5 h	Model assumption; sensitivity values 0.25 and 1.0 h
Escort time, hospital pathway	5.15 h	Proxy based on X-ray-only international ED/pathway LOS [6], not directly measured caregiver time; Greek-informed scenarios 3.42, 4.5 and 6.2 h; stress-test range 2-8 h
Value of escort time	5.31 €/h	Opportunity cost, Greek minimum wage [30]; sensitivity values €0 and €12/h
Cost year	2026	All costs in 2026 euros

## Results

Base-case societal analysis

For the home pathway: $C_{\mathrm{home}} = 110 + (0.5 \times 5.31) = &euro;112.66$; for the hospital pathway:​​​​​​​ $C_{\mathrm{hospital}} = 8.10 + 100 + (5.15 \times 5.31) = &euro;135.45$. Therefore, * Δ C = 112.66 - 135.45 = -&euro;22.79 *.

In the base-case scenario of the limited societal perspective defined in this model, home two-view chest radiography was €22.79 less expensive per diagnostic episode than the hospital pathway. The base-case societal results are summarized in Table 2.

**Table 2:** Base-case societal analysis

Strategy	Cost components	Total cost
Home-based chest radiography	Home X-ray + escort time	112.66 €
Hospital-based chest radiography	Hospital X-ray + transport + escort time	135.45 €
Incremental cost	Home - Hospital	-22.79 €

Healthcare system perspective

From the healthcare system perspective, two scenarios were presented. In the first scenario, the payer was assumed to cover only the examination. In the second scenario, the payer was assumed to cover both the examination and transport. The healthcare system perspective results are presented in Table 3.

**Table 3:** Payer perspective

Scenario	Home pathway	Hospital pathway	Difference
Payer covers examination only	110.00 €	8.10 €	+101.90 €
Payer covers examination and transport	110.00 €	108.10 €	+1.90 €

From a narrow healthcare system perspective, if only the examination is reimbursed, home radiography appears more expensive. When transportation is factored in, the difference between the two strategies narrows considerably.

Sensitivity and threshold analyses

The one-way sensitivity and threshold analyses are presented in Table 4.

**Table 4:** One-way sensitivity and threshold analyses

Parameter	Alternative value	Incremental cost	Threshold for home pathway to remain cost-saving
Home X-ray cost	50 €	-82.79 €	<132.79 €
Home X-ray cost	90 €	-42.79 €	<132.79 €
Home X-ray cost	140 €	7.21 €	<132.79 €
Transport cost	90 €	-12.79 €	>77.21 €
Transport cost	130 €	-52.79 €	>77.21 €
Hospital escort time	2 h	-6.07 €	>0.86 h
Hospital escort time	8 h	-37.93 €	>0.86 h
Escort hourly value	12 €/h	-53.90 €	>0.41 €/h
Escort hourly value	0 €/h	1.90 €	>0.41 €/h
Hospital X-ray cost	20 €	-34.69 €	Not critical
Home escort time	0.25 h	-24.12 €	<4.79 h
Home escort time	1.0 h	-20.14 €	<4.79 h

The home strategy remained less expensive in most model variants. The hospital pathway became less expensive when the home X-ray price was increased to €140 and when the economic value of attendant time was set at €0/hour, with all other parameters held constant. Importantly, widening hospital escort time downward to two hours did not reverse the result; the home pathway remained €6.07 less expensive. The tornado diagram is shown in Figure 2.

**Figure 2:**
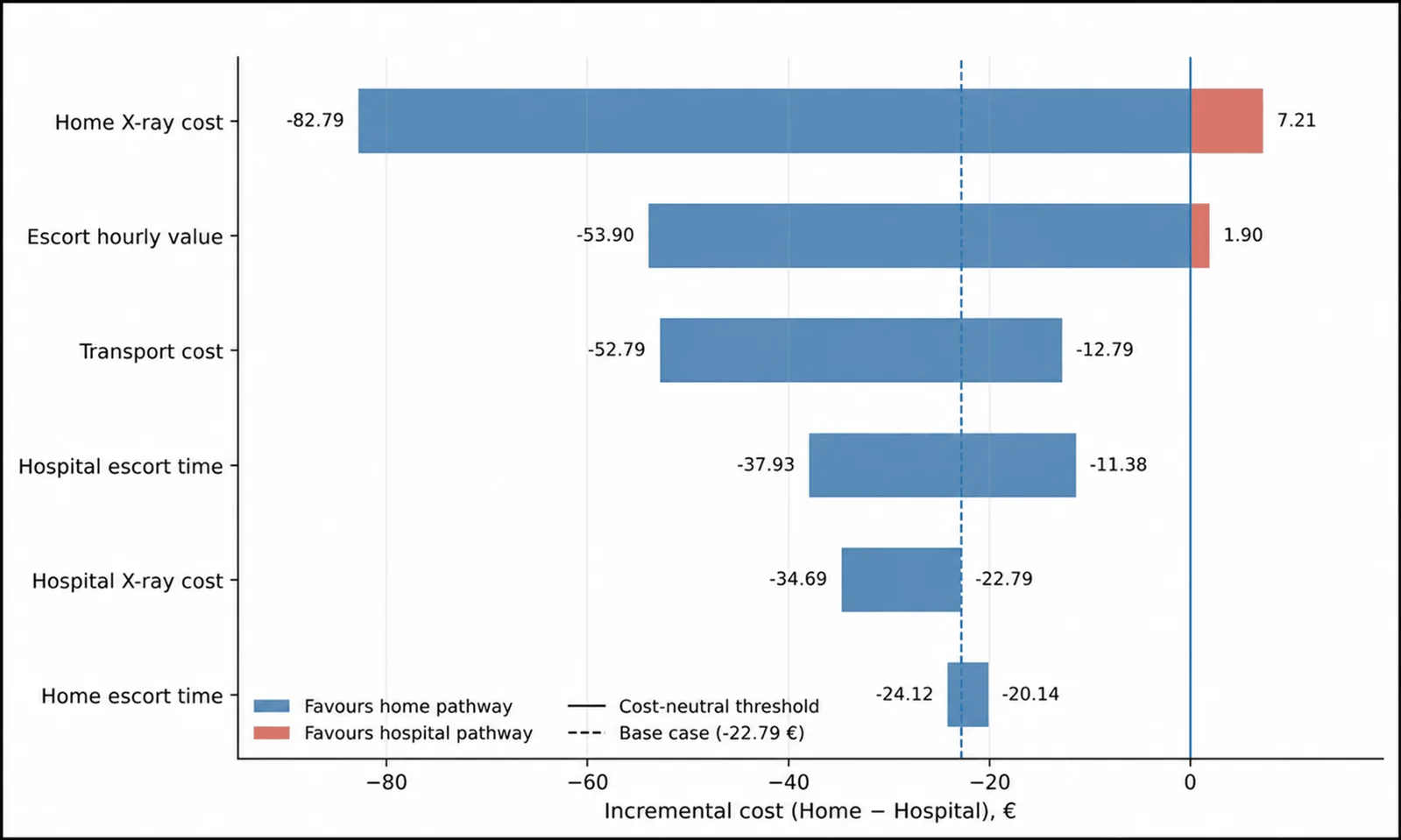
Tornado diagram for the one-way sensitivity analysis Bars show the range of incremental costs after varying each parameter. Negative values favor the home pathway, whereas positive values favor the hospital pathway. The dashed vertical line indicates the base-case incremental cost (-€22.79), and the solid vertical line indicates the cost-neutral threshold.

Scenario analyses

The scenario analyses are presented in Table 5.

**Table 5:** Scenario analyses

Scenario	Assumptions	Home cost	Hospital cost	Difference
Price-data robustness	Lowest “from €50” observation excluded; revised median home X-ray €115	€117.66	€135.45	-€17.79
Short hospital pathway stress test	Hospital escort time 2 h	€112.66	€118.72	-€6.07
Greek ED waiting + care scenario	Hospital escort time 3.42 h [8]	€112.66	€126.28	-€13.62
Greek multicenter ED LOS - lower	Hospital escort time 4.5 h [9]	€112.66	€132.00	-€19.34
Greek multicenter ED LOS - upper	Hospital escort time 6.2 h [9]	€112.66	€141.02	-€28.37
Difficult hospital pathway	Transport €130; escort time 8 h	€112.66	€180.58	-€67.93
Easier hospital pathway	Transport €90; escort time 3 h	€112.66	€114.03	-€1.38

The direction of the result remained in favor of the home pathway across the Greek hospital-time scenarios and after excluding the lowest starting-price observation. Even under a two-hour hospital-attendant-time stress test, home radiography remained less costly by €6.07. The easier hospital-pathway scenario combining €90 transport and three hours of attendant time remained close to cost-neutral (-€1.38).

The conservative stress test for technically unsuccessful home examinations is presented in Table 6.

**Table 6:** Conservative stress test for technically unsuccessful home examinations followed by the full hospital pathway The break-even probability is approximately 16.8%.

Hypothetical home failure/referral probability	Expected cost of initiating home pathway	Hospital pathway cost	Incremental cost
0%	€112.66	€135.45	-22.79 €
5%	€119.43	€135.45	-16.02 €
10%	€126.20	€135.45	-9.25 €
15%	€132.97	€135.45	-2.47 €
20%	€139.74	€135.45	+4.30 €

In this stress test, the expected cost of the home strategy remained below the hospital pathway at 5%, 10%, and 15% failure/referral probabilities and became higher at 20%. The cost-neutral probability was 16.8%. These values should not be interpreted as estimated clinical failure rates; they quantify how large the probability of a consequential home-to-hospital failure event would need to be, under the conservative costing assumption used, to reverse the base-case economic result.

## Discussion

The present modeled analysis shows that home two-view chest radiography may be less costly than the hospital pathway for appropriately selected, clinically stable patients with limited mobility when assessed from a limited societal perspective. In the base-case scenario, the home strategy was €22.79 less expensive per completed diagnostic episode.

This interpretation applies only to patients for whom the sole anticipated benefit of hospital transport is completion of radiography. It does not apply to patients with acute clinical deterioration or a possible need for additional diagnostic evaluation or therapeutic management in the ED.

This finding does not arise because home X-ray is less expensive as a standalone test. By contrast, the price of the home service is substantially higher than the EOPYY reimbursement tariff used as a proxy for the hospital X-ray cost. The economic advantage emerges when the examination is considered within the overall pathway, i.e., when patient transport and the economic value of attendant time are taken into account. Dependence on the latter parameter is substantial: when attendant time was assigned no economic value, the home pathway became marginally more expensive by €1.90.

Eleven operationally comparable observations produced a median of €110 (IQR €100-€120; range €50-€140). Because the lowest observation was advertised as a starting price, we also excluded it in a robustness analysis; the resulting median increased to €115, yet the home pathway remained €17.79 less expensive. Conversely, the €140 upper-market scenario made the home pathway €7.21 more expensive. Under the base-case assumptions, home radiography remained cost-saving while the price of the home two-view examination was below approximately €132.79 per episode. The model also yields a transport-cost threshold above €77.21 and an attendant-time-value threshold above €0.41/hour; these are mathematical thresholds conditional on the other model inputs and should not be interpreted as clinically validated economic cut-offs. These findings reinforce that the home-service price is a major model driver and should not be interpreted as a fixed national tariff.

The 5.15-hour base-case value remains an international estimate but is closely matched to the modeled X-ray-only pathway [6]. Greek real-world ED studies report substantial but heterogeneous times: approximately 3.42 hours when the mean waiting and care components in the Nikaia study are combined [8], and average ED length of stay of 4.5-6.2 hours across four Greek public EDs [9]. Because these Greek populations are broader than the modeled X-ray-only cohort, they were used as scenario values rather than direct replacements. The home pathway remained less costly across these scenarios and even at a two-hour stress-test value; nevertheless, the zero-value-of-attendant-time scenario still reversed the direction of the result. The societal conclusion therefore depends on both the duration and the monetary valuation of attendant time.

The findings are consistent with international studies showing that mobile radiography can reduce hospital resource use, transport, and ED use [4,10,12]. A scoping review by Costa et al. places home radiography within the broader field of home diagnostic services, where successful implementation depends on equipment portability, appointment organization, secure data transfer, and integration into the diagnostic pathway [1].

The study by Eade et al. is particularly relevant to the present analysis because it concerns the real-world home deployment of mobile radiography in a regional setting. Their service evaluation reported an overall image-repeat rate of 10.3% [4]. However, an image repeated during the same attendance is not equivalent to a failed diagnostic episode, a second home visit, or hospital referral. We therefore did not use that image-repeat rate as a home-pathway failure probability. Instead, the revised model uses a hypothetical conservative stress test in which an unsuccessful home examination incurs both the initial home cost and the full subsequent hospital-pathway cost. Under this assumption, the break-even home failure/referral probability is approximately 16.8%.

The effective implementation of home radiography requires reliable digital image management and the capacity for remote consultation. Teleradiology is an established component of modern radiology practice, allowing images to be transmitted and radiological advice to be provided outside the acquisition site. In this context, home radiography is not an isolated service but part of a broader digital diagnostic pathway linking image acquisition at home, secure image transfer, and remote radiological consultation [14].

The choice of perspective is critical. From a narrow payer perspective, if only hospital X-ray reimbursement is considered, home testing appears more expensive. From the limited societal perspective of the model, in which transportation and the economic value of attendant time are included, the result is reversed in favor of the home strategy. Therefore, the conclusions should not be interpreted independently of the cost categories included in the analysis. This has implications for the formulation of reimbursement policies and the development of home care services.

Patient acceptance is also a relevant contextual dimension. Toppenberg et al. reported higher satisfaction with mobile radiography compared with hospital radiography [5]. However, patient satisfaction and other patient-reported outcomes were not measured in the present study and were not used to substantiate the modeled cost-saving result; these findings are drawn from external literature only.

Beyond cost, external studies suggest that mobile radiography can improve access to diagnostic services. Kjelle et al. showed that the availability of a mobile radiography service in nursing homes can increase the use of diagnostic imaging by nursing home residents, thereby partially reducing the access gap compared with the general population [11]. The inclusion of home radiography in organized care models should, however, be accompanied by clear eligibility criteria. Kobes et al. showed that the development of multidisciplinary guidelines for mobile radiography can reduce the number of inappropriate mobile chest X-rays [31]. These access and implementation findings provide context but are not outcomes generated by the present economic model.

This analysis did not quantify intangible costs such as stress, mental burden, discomfort, or potential disorganization of elderly and vulnerable patients during transport to and time spent in a hospital setting. Because these elements were not quantified, they were not used to substantiate the cost-saving conclusion but represent additional dimensions that could be considered in a future cost-consequence analysis.

Limitations

A key limitation is the use of EOPYY reimbursement rates as a proxy for the cost of hospital two-view chest radiography. The full operating cost of a radiology service in a public hospital, particularly during on-call periods, may differ from the official reimbursement tariff because it includes staff, supplies, equipment, depreciation, administrative support, and overhead. However, in the absence of available Greek procedure-level micro-costing data for public hospitals, use of the reimbursement tariff was considered a transparent and available approach. In addition, the model combines different economic metrics-reimbursement tariffs, publicly posted commercial charges, and opportunity-cost valuation of time-that do not represent equivalent measures of full resource cost. This heterogeneity directly affects the interpretation of the economic result. Accordingly, the study should be regarded as an exploratory pathway-level cost comparison based on available Greek price, reimbursement, and opportunity-cost proxies rather than as a full economic resource-costing study.

Second, the base-case scenario involves a hospital pathway through a public hospital ED rather than a visit to a private diagnostic center or direct hospital-radiology service. The ED comparator is an explicit organizational scenario and is not intended to represent a universal Greek pathway. A direct radiology or outpatient diagnostic pathway, where available, may involve shorter waiting and attendant time and could reduce the modeled advantage of home radiography. Conversely, because no separate ED assessment, staffing, laboratory, treatment, or other emergency-department costs were included, the hospital-pathway cost may be underestimated if such resources are actually consumed in practice. The comparator should therefore be interpreted solely for clinically stable patients transferred for radiography rather than for patients requiring a full emergency evaluation.

Third, home-radiography prices remain market charges rather than audited resource costs. The revised analysis substantially improves transparency by using explicit comparability criteria and a provider-level supplementary table, but some provider information was obtained from online booking or direct provider confirmation rather than a standardized tariff. Geographic, out-of-hours, and distance-related surcharges may also apply. In addition, the transport-price observations are based on private ambulance providers in Attica, while home-radiography availability and pricing may differ between urban and rural areas, mainland and island settings, and according to travel distance and local service infrastructure. The €22.79 base-case difference should therefore not be interpreted as a nationally representative Greek estimate. Importantly, excluding the lowest starting-price observation increased the median home-radiography price to €115 without reversing the result.

Fourth, the 5.15-hour hospital attendant-time parameter is based on international rather than Greek X-ray-only data and is derived from patient ED/pathway length of stay rather than direct measurement of caregiver time. The model uses this value as a proxy for attendant-accompanied in-hospital time; ambulance travel time and healthcare-professional time are not separately monetized. Greek ED studies were identified and incorporated into scenario analyses, but their populations are broader than the modeled comparator and do not directly measure a transport-plus-radiography-only pathway in mobility-limited patients. The home strategy remained less costly across Greek-informed time scenarios and a wider two- to eight-hour range, but the conclusion depends substantially on both the duration and the economic valuation of attendant time; assigning a value of zero to attendant time reverses the direction of the limited-societal-perspective result.

Fifth, the base-case model assumes successful completion of a diagnostic-quality examination in both pathways and does not assign an empirical probability to technically unsuccessful home imaging followed by repeat attendance or hospital referral. Published mobile-radiography image-repeat rates cannot be assumed to represent complete diagnostic failure. The added stress test therefore evaluates hypothetical home-to-hospital failure/referral probabilities rather than claiming an evidence-based rate. Under the deliberately conservative assumption that a failed home episode incurs both the home and full hospital costs, the break-even probability is approximately 16.8%.

Finally, the analysis remains model-based, does not include prospective Greek patient-level resource-use data, and did not include a probabilistic sensitivity analysis. Probability distributions for several influential inputs - particularly service prices, pathway times, and consequential home-to-hospital failure rates - are not established from representative Greek samples. The findings should therefore be considered exploratory and model-dependent, and should be confirmed in future prospective service evaluations or time-and-motion studies.

## Conclusions

Under the specific assumptions of the model, home two-view chest radiography may be an economically comparable or potentially cost-saving alternative to the modeled hospital pathway for appropriately selected, clinically stable patients with limited mobility when the analysis is performed from a limited societal perspective and transportation costs and the economic value of attendant time are taken into account. In the base-case scenario, the home strategy was €22.79 less expensive per completed diagnostic episode; this value is an illustrative model estimate rather than a nationally representative Greek cost difference.

The economic result depends mainly on the price of the home X-ray, transport costs, and the duration and valuation of attendant time. Revised Greek-informed hospital-time scenarios and a conservative technical-failure/referral stress test did not overturn the base-case conclusion within the ranges examined, but alternative assumptions can narrow or reverse the cost difference. The findings should therefore be interpreted as exploratory, model-dependent evidence rather than definitive proof of economic superiority. Home radiography should not be considered a substitute for urgent hospital evaluation, and prospective Greek service evaluations using patient-level clinical, pathway, and resource-use data are needed before reimbursement or implementation decisions are made.

## Appendices

Appendix 1

**Table 7:** Publicly advertised Greek prices for home-based digital two-view radiography and service characteristics Price observations were used to inform the base-case home radiography cost. Services were included when the available provider information, supplemented where necessary by direct provider/author confirmation, supported a home-based digital examination with two projections/two acquisitions and radiologist interpretation/reporting. Prices are publicly advertised charges rather than audited economic resource costs.

Provider	Advertised price (€)	Digital/two-view protocol	Home attendance/travel	Radiologist report	Image/result delivery	Geographic coverage	Additional charges/conditions	Evidence basis
Kitrinos Stavros	50	Yes	Yes	Yes	Electronic delivery reported	Attica	Starting/advertised price; package conditions may vary	Provider website + additional confirmation
Aktinoapikonisi Express	90	Yes - F/Pr	Yes	Yes	DICOM CD and electronic delivery	Attica	Not stated on the cited page	Provider website
24Xray	100	Yes - up to 2 digital images	Yes	Yes	Printed report/digital image delivery	Attica	Out-of-hours/area conditions may apply	Provider website
TreatMED	100	Yes	Yes	Yes	Electronic image/result transmission	Attica	May vary by service/location	Public price list + additional confirmation
Instahealth	100	Yes	Yes	Yes	Electronic diagnosis/result delivery	Attica	Booking-specific conditions may apply	Provider website + online booking flow
Oikoiatriki	110	Yes - 2 views (F + profile)	Yes	Yes	Digital images and report	Attica	Geographic/service conditions may apply	Provider website
HomeXRays	120	Yes - F+Pr	Yes	Yes	Electronic delivery; CD available	Attica	Weekend/night and remote-area surcharges may apply	Provider website
Doctor Home Care	120	Yes - 2 digital acquisitions	Yes	Yes	Images/report; electronic transmission available	Athens/Attica	Geographic conditions may apply	Provider website
Nosileia247	120	Yes - 2 digital acquisitions	Yes	Yes	Images/report; electronic transmission available	Attica	Not clearly stated on cited page	Provider website
MED X RAYS	120	Yes - F+P	Yes	Yes	Radiologist report; electronic delivery available	Athens/Piraeus and wider Attica	Area-dependent pricing/surcharges	Provider website
SOS IATROI	140	Yes - first 2 digital acquisitions	Yes	Yes	Signed report, CD; electronic transmission available	Attica/Greece service network	Higher out-of-hours/weekend charges may apply	Provider website
